# In Vivo Risk Assessment of Yellow Fever Virus Transmission Through Breastfeeding, and Mechanistic Insights

**DOI:** 10.1093/infdis/jiaf637

**Published:** 2025-12-17

**Authors:** Jeanne Pascard, Sophie Desgraupes, Aurélie Chiche, Patricia Jeannin, Rebecca Kanaan, Antoine Gessain, Han Li, Pierre-Emmanuel Ceccaldi, Aurore Vidy

**Affiliations:** Institut Pasteur, Université Paris-Cité, Epidemiology and Pathophysiology of Oncogenic Viruses Unit, Virology Department, Paris, France; Institut Pasteur, Université Paris-Cité, Epidemiology and Pathophysiology of Oncogenic Viruses Unit, Virology Department, Paris, France; Institut Pasteur, Centre National de la Recherche Scientifique, Cellular Plasticity and Age-Related Pathologies Group, Developmental and Stem Cell Biology Department, unité mixte de recherche 3738, Paris, France; Institut Pasteur, Université Paris-Cité, Epidemiology and Pathophysiology of Oncogenic Viruses Unit, Virology Department, Paris, France; Institut Pasteur, Université Paris-Cité, Epidemiology and Pathophysiology of Oncogenic Viruses Unit, Virology Department, Paris, France; Institut Pasteur, Université Paris-Cité, Epidemiology and Pathophysiology of Oncogenic Viruses Unit, Virology Department, Paris, France; Institut Pasteur, Centre National de la Recherche Scientifique, Cellular Plasticity and Age-Related Pathologies Group, Developmental and Stem Cell Biology Department, unité mixte de recherche 3738, Paris, France; Institut Pasteur, Université Paris-Cité, Epidemiology and Pathophysiology of Oncogenic Viruses Unit, Virology Department, Paris, France; Institut Pasteur, Université Paris-Cité, Epidemiology and Pathophysiology of Oncogenic Viruses Unit, Virology Department, Paris, France

**Keywords:** yellow fever virus, transmission via breastfeeding, mammary gland, breast milk, intestinal epithelium crossing

## Abstract

**Background:**

Yellow fever virus (YFV), a mosquito-borne orthoflavivirus, remains a significant public health concern, especially in regions with low vaccine coverage. Since 2010, yellow fever vaccination is not recommended for breastfeeding women due to reported cases of vaccine strain transmission through breast milk causing neonatal meningoencephalitis. However, breastfeeding transmission efficiency of the vaccine strains remains unknown, and wild-type strain transmission has been suggested following viral RNA detection in milk. Obtaining direct evidence of breastfeeding-related transmission in humans is challenging as vector-borne exposure confounds analyses, making animal models essential for assessing this risk.

**Methods:**

We used an A129 mouse model to investigate YFV transmission via breastfeeding for wild-type and vaccine strains, and human epithelial in vitro models to explore mechanisms of mammary and intestinal barrier crossing.

**Results:**

Wild-type and vaccine strains spread to mammary glands, targeting mainly stromal and immune cells, and are excreted into milk as free and cell-associated virus. In vitro, mammary epithelial cells also support infection, suggesting 2 mechanisms of epithelial crossing. Neonates are susceptible to oral infection, showing higher infection rates for wild-type virus but evidence of neuroinvasion for both strains. These strains infect and cross an in vitro human intestinal barrier model, suggesting this epithelium as a potential viral entry site for neonates. Finally, the virus can be transmitted from infected dams to suckling pups via breastfeeding, though rarely.

**Conclusions:**

This study demonstrates YFV transmission through breastfeeding in an animal model and supports the biological plausibility of this route, highlighting its potential among YFV transmission risks.

## Teaser

Yellow fever virus can be transmitted through breastfeeding in a mouse model, albeit with limited efficiency. Both vaccine and wild-type strains reach the mammary glands and cross the neonatal intestinal barrier, supporting breastfeeding as a possible route of transmission.

## Graphical Abstract

**Graphical Abstract:**
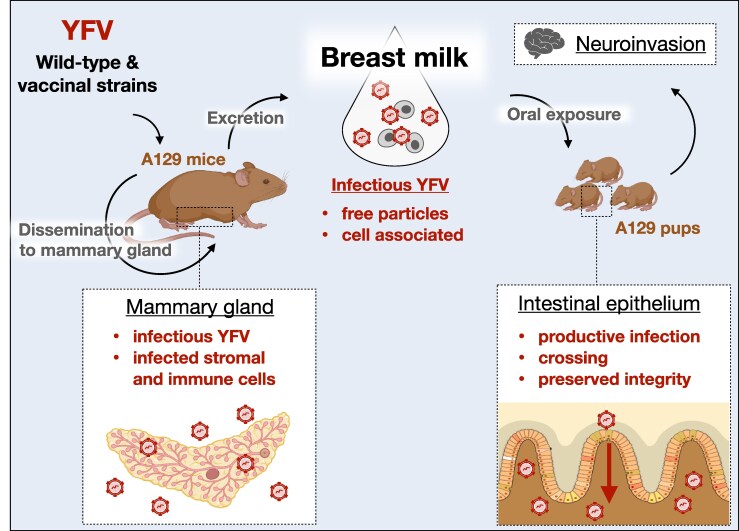

Yellow fever virus (YFV) is a mosquito-borne orthoflavivirus that causes a potentially fatal hemorrhagic disease, with approximately 200 000 cases and 30 000 deaths annually worldwide [1]. Clinical manifestations range from mild symptoms to severe jaundice and hemorrhagic fever, with fatality rates up to 60% in severe cases [2]. Despite the availability of a safe and effective live-attenuated vaccine (17D), YFV remains endemic in 44 countries, and outbreaks continue due to insufficient immunization coverage [3]. Currently, 3 vaccine substrains are used worldwide: 17D-204, 17DD, and 17D-213 [4].

In addition to mosquito-borne transmission, YFV has been implicated in nonvectorial routes such as blood transfusion [5, 6], organ transplantation [6], and vertical transmission [7–12]. Notably, several cases of breastfeeding transmission involving YFV vaccine strains have been reported. In each instance, exclusively breastfed infants developed meningoencephalitis shortly after maternal vaccination [9–12]. Detection of vaccine RNA or YFV-specific immunoglobulin M in the infants’ serum and/or cerebrospinal fluid provided compelling evidence of transmission via breastfeeding [9–11]. Consequently, since 2010, the US Centers for Disease Control and Prevention recommends against vaccinating breastfeeding women, except for epidemics or unavoidable travel to endemic areas [13].

Although less documented, wild-type YFV transmission via breastfeeding remains plausible. During Brazil's 2016–2018 outbreak, YFV RNA was detected in breast milk of an infected mother whose infant also developed symptoms [14], raising concern about this potential, but often underestimated, transmission route. However, establishing breastfeeding transmission of mosquito-borne viruses is difficult in endemic areas, where the omnipresence of vectors makes it nearly impossible to exclude mosquito-borne transmission.

To address this challenge, criteria adapted from Koch postulates have been proposed [15]. These include epidemiological evidence of breastfed infant infection, detection of infectious virus in breast milk, exclusion of alternative transmission routes, and reproduction of transmission via oral or breastfeeding routes in animal models. While epidemiological data are informative, animal models are essential to confirm causality under controlled conditions. Importantly, transmission of the YFV 17D vaccine strain through breastfeeding is considered proven, as this attenuated strain cannot be transmitted by mosquitoes [16], thereby excluding vectorial transmission and strengthening the causal link.

Using these criteria, breastfeeding transmission has been confirmed for 3 human viruses: human T-cell lymphotropic virus type 1 (HTLV-1), human immunodeficiency virus (HIV), and human cytomegalovirus (CMV) [15], and is suspected for several arboviruses. Infant infections have been reported following maternal infection with chikungunya virus, dengue virus (DENV), West Nile virus, and Zika virus (ZIKV), with viral genomes from all 4—and infectious DENV and ZIKV particles—detected in breast milk [17].

In this study, we used in vivo (mouse) and in vitro (human cells) models to investigate YFV transmission to neonates via oral exposure and breastfeeding. We demonstrate that both vaccine and wild-type strains infect the mammary gland, cross the mammary epithelium, and are excreted in murine milk as infectious free particles and cell-associated virus. In vivo, YFV primarily targets mammary stromal and immune cells; however, human mammary epithelial cells are also permissive to infection, suggesting 2 epithelial-crossing mechanisms in mammary glands: infected immune cell transmigration (“Trojan horse” strategy), and epithelial infection with viral release. We also show that neonatal mice are susceptible to YFV via the oral route, with neuroinvasion following intragastric exposure. Using a human intestinal epithelium model, we demonstrate that both strains infect and cross this barrier without disrupting its integrity. Finally, we confirm YFV transmission to suckling pups via breastfeeding—albeit infrequently—validating this route of transmission for YFV.

## MATERIALS AND METHODS

### Animal Model

Experiments used the A129 mouse model (129S2/SvPas-Ifnar1^tm1Agt^), deficient in the interferon-α/β receptor (IFNAR1^−^/^−^). Mice were housed and bred in the Institut Pasteur (Paris, France), in animal facilities accredited by the French Ministry of Agriculture for breeding and performing experiments on live rodents (authorization A75150101).

### Virus Strains

Three YFV strains were used: wild-type strains Asibi (GenBank: AY640589.1) and Dakar/HD1279 (GenBank: MN106242.1), and vaccine strain 17D-204 (GenBank: MN708488.1).

### Mouse Infection

Nonlactating females were subcutaneously inoculated with 1–3 × 10^5^ plaque-forming units (PFU) of YFV (Asibi, Dakar/HD1279, or 17D-204 strains). Blood and mammary glands were collected at various days postinoculation (dpi). Lactating females received subcutaneously 1–3 × 10^5^ PFU of YFV (Asibi or 17D-204). Maternal blood, breast milk, and mammary glands were collected 4–6 dpi [18]. For breastfeeding-mediated transmission experiments, pups’ blood and organs were collected 7–9 dpi. For oral infection of neonatal mice, A129 pups received 2 × 10^4^ to 1 × 10^5^ PFU of YFV (Asibi or 17D-204) by the intragastric route. Blood and brains were collected at 6 dpi. Protocols and animal monitoring are detailed in the Supplementary Material.

### Ethics Statement

Animal experiments complied with French and European regulations on care and protection of laboratory animals (EC Directive 2010/63, French Law 2013-118, 6 February 2013). All experiments were approved by the Ethics Committee number 89 and registered by the French “Ministère de l’Enseignement Supérieur, de la Recherche et de l’Innovation” (MESRI) under the reference APAFIS#31829–2021052812549296 (date of approval: 31 May 2021). Use of genetically modified mice (A129) was approved by the institutional review boards and the MESRI under the reference number 9319 (date of approval: 6 December 2021).

Full protocols are available in the Supplementary Material.

## RESULTS

### YFV Disseminates to the Mammary Glands of Nonlactating and Lactating A129 Mice

**Figure 1.**
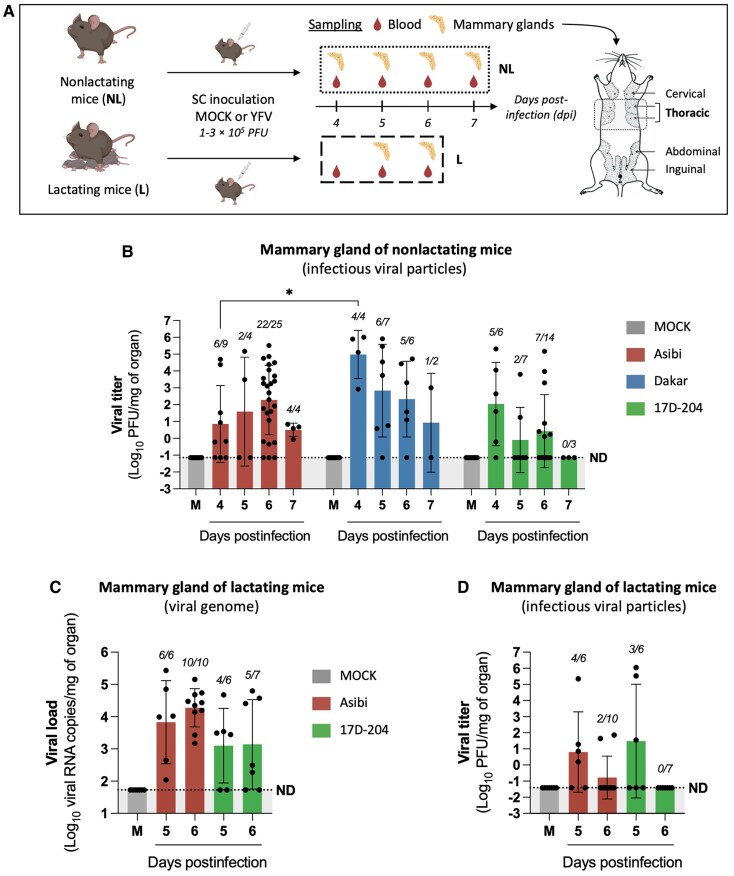
Yellow fever virus (YFV) disseminates to the mammary glands of nonlactating and lactating A129 mice. *A*, Nonlactating (6–22 weeks old) and lactating (10–22 weeks old) A129 mice were either mock-inoculated (MOCK) or inoculated with YFV via subcutaneous (SC) injection. Nonlactating females were inoculated with phosphate-buffered saline (PBS) (MOCK, n = 16) or with 1 to 3 × 10^5^ plaque-forming units (PFU) of the Asibi (n = 42), Dakar/HD1279 (n = 19), or 17D-204 strains (n = 30) of YFV (7 independent experiments). Lactating females were inoculated 1–13 days postpartum with PBS (MOCK, n = 7) or with 1–3 × 10^5^ PFU of the Asibi (n = 16) or 17D-204 strains (n = 13) (5 independent experiments). At various days postinfection (dpi), blood samples and thoracic mammary glands were collected to assess mice infection and mammary gland dissemination, respectively (illustration created with BioRender). *B*, YFV dissemination to thoracic mammary glands of nonlactating mice was assessed by plaque assay on Vero cells to detect infectious viral particles. *C* and *D*, Viral dissemination to thoracic mammary glands of lactating mice was assessed by reverse-transcription quantitative polymerase chain reaction using NS3-specific primers to determine the presence of viral RNA genome (*C*) and by plaque assay on Vero cells (*D*). The number of infected mammary glands relative to the total number of tested mammary glands is shown in italics above each time point. Results are expressed as the mean ± standard deviation. The dashed lines indicate the limit of detection or specificity (determined from MOCK control conditions), below which values were considered nonspecific or not detected. The highest of these 2 thresholds was chosen as the final limit (ND). Statistical tests: Kruskal–Wallis test with Dunn multiple comparisons was used all in panels, comparing means values between all strains at all time points. *P < .05. Only statistically significant differences (*P* < .05) are indicated.

To investigate the early steps of YFV transmission via breastfeeding, A129 mice, a well-established model of YFV infection [19], were used to assess viral dissemination to mammary glands and subsequent excretion into breast milk. Infection kinetics were first evaluated in nonlactating females for ethical and practical reasons, with key findings later validated in lactating mice. Mice were subcutaneously inoculated with wild-type Asibi or Dakar/HD1279 strains, or vaccine 17D-204 strain (1–3 × 10^5^ PFU), modeling a local infection as occurs following mosquito bite. Clinical signs, weight, and viremia were monitored, and mammary glands were collected at various days postinfection (dpi) (Figure 1*A*). No mortality was observed. Only wild-type strains induced mild symptoms and limited weight loss (∼5% for Dakar), while infection with 17D-204 strain remained asymptomatic (Supplementary Figure 1*A* and 1*B*). Viremia was confirmed in all groups by the presence of viral RNA (vRNA) in the plasma, except in three 17D-204 cases, where infection was confirmed in the spleen (Supplementary Figure 1*C*). In nonlactating mice (6–22 weeks old), infectious virus was detected by plaque assay in mammary glands for all strains, peaking between 4 and 6 dpi with notable interindividual variation (Figure 1*B*), including within an age-matched group (8–12 weeks; Supplementary Figure 1*E*). Titers declined by 7 dpi, suggesting limited persistence in this tissue. When pooling all time points, mammary infection was significantly more frequent with wild-type Asibi (73.8%) and Dakar (84.2%) than with 17D-204 (46.7%) (Supplementary Figure 1*D*-a), suggesting limited dissemination of the vaccine strain. Using the identified peak infection times (5–6 dpi), presence of vRNA and infectious particles in mammary glands of lactating females was confirmed (Figure 1*C* and 1*D*), with marked interindividual variability. Reverse-transcription quantitative polymerase chain reaction (RT-qPCR) detection was significantly more frequent in Asibi-infected mice (100%) than in 17D-204–infected mice (69.2%) (Supplementary Figure 1*D*-b), further supporting reduced dissemination of the vaccine strain. Notably, infectious virus was detected less frequently in lactating than in nonlactating glands, likely due to technical challenges in viral quantification, as milk and tissue remodeling may reduce detection sensitivity. These results show that both wild-type and vaccine YFV strains can reach the mammary glands in mice under both lactating and nonlactating conditions.

### YFV Infectious Virus Is Released Into Murine Breast Milk, as Free Viral Particles and Cell-Associated Virus

**Figure 2.**
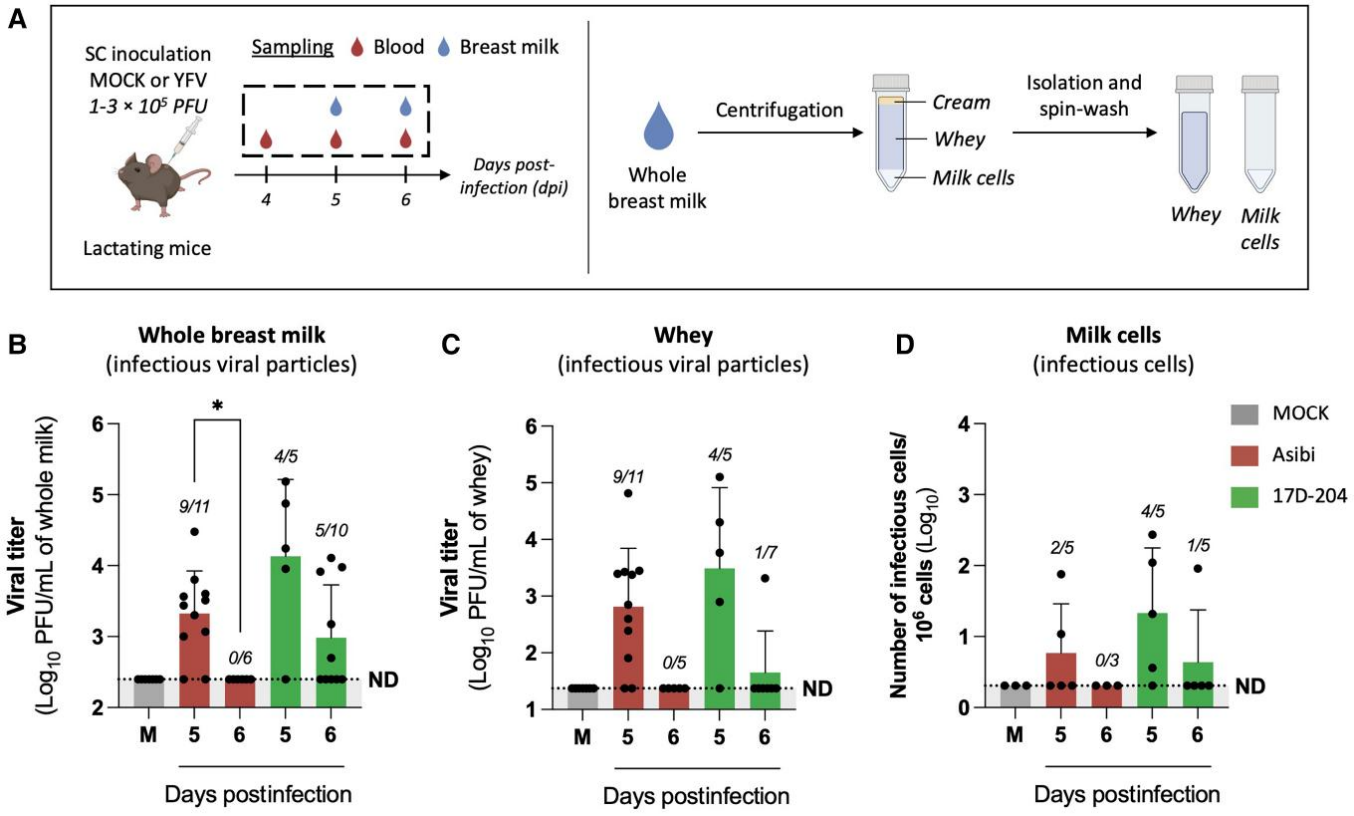
Yellow fever virus (YFV) infectious virus is released into murine breast milk, both as free viral particles and cell-associated virus. *A*, Lactating A129 mice (10–22 weeks old) were either MOCK-inoculated (n = 7) or inoculated with YFV at 1–12 days postpartum via subcutaneous (SC) injection of 1–3 × 10^5^ plaque-forming units (PFU) of the Asibi (n = 17) or 17D-204 (n = 15) strains of YFV (6 independent experiments). Four to 6 days after infection (dpi), blood and breast milk samples were collected. For some milk samples, a portion was centrifuged to separate cream, whey, and cells. Whey was further clarified by 3 centrifugations to enhance purity, while the cell pellet underwent 3 wash–spin cycles in PBS to remove free viral particles (illustration created with BioRender). *B* and *C*, Detection of YFV in whole breast milk (*B*) and whey (*C*) was assessed by plaque assay on Vero cells to determine the presence of infectious viral particles. *D*, Detection of infectious cell–associated virus in the milk cell pellet was performed by infectious center assay. The number of infected samples relative to the total number of tested samples per condition is shown in italics above each time point. Results are expressed as the mean ± standard deviation. The dashed lines indicate the limit of detection, below which values were considered as not detected (ND). Statistical tests: Kruskal–Wallis test with Dunn multiple comparisons was used in all panels, comparing means values between all strains at all time points. *P < .05. Only statistically significant differences (*P* < .05) are indicated.

To investigate viral excretion into milk, lactating A129 mice were inoculated with YFV Asibi or 17D-204 strains via the subcutaneous route (Figure 2*A*), and successful infection was confirmed in plasma (Supplementary Figure 2*A*) or spleen (Supplementary Figure 1*C*-c). Viral RNA was detected in breast milk collected at 5–6 dpi for both strains in 80%–90% of animals (Supplementary Figure 2*D*-a), ranging from 2 × 10^3^ to 7.1 × 10^5^ (Asibi) and 2.5 × 10^4^ to 4.7 × 10^6^ vRNA copies/mL (17D-204) (Supplementary Figure 2*C*-a), reflecting a high rate of viral shedding into milk, without correlation between plasma and milk viral loads (Supplementary Figure 2*B*). Infectious virus was also detected, with higher titers at 5 dpi—generally greater for 17D-204 (up to 1.5 × 10^5^ PFU/mL) than Asibi (up to 3 × 10^4^ PFU/mL)—though differences were not statistically significant (Figure 2*B*). Titers declined by 6 dpi, with infectious virus undetectable in the Asibi group and lower detection in 17D-204 samples. To characterize the form of virus in milk, murine milk samples were fractionated by centrifugation into cream, whey, and cellular components (Figure 2*A*). vRNA was consistently detected in whey and purified milk cells (Supplementary Figure 2*C*-b and 2*C*-c), and infectious virus was found in whey (Figure 2*C*) and in milk cells after co-culture with permissive cells (Figure 2*D*), indicating the presence of both free viral particles and cell-associated infectious virus in breast milk. These findings demonstrate that both wild-type and vaccine YFV strains are excreted into breast milk as free and cell-associated infectious virus. No strain-specific differences were observed in viral load, infectious titer, or the proportion of positive milk samples (Supplementary Figure 2*D*), which contrasts with the dissemination patterns seen in mammary tissue. This viral excretion into breast milk requires crossing the mammary epithelium, via mechanisms yet to be elucidated.

### YFV Infects Mainly Immune and Stromal Cells in the Mammary Glands of Nonlactating Mice In Vivo

**Figure 3.**
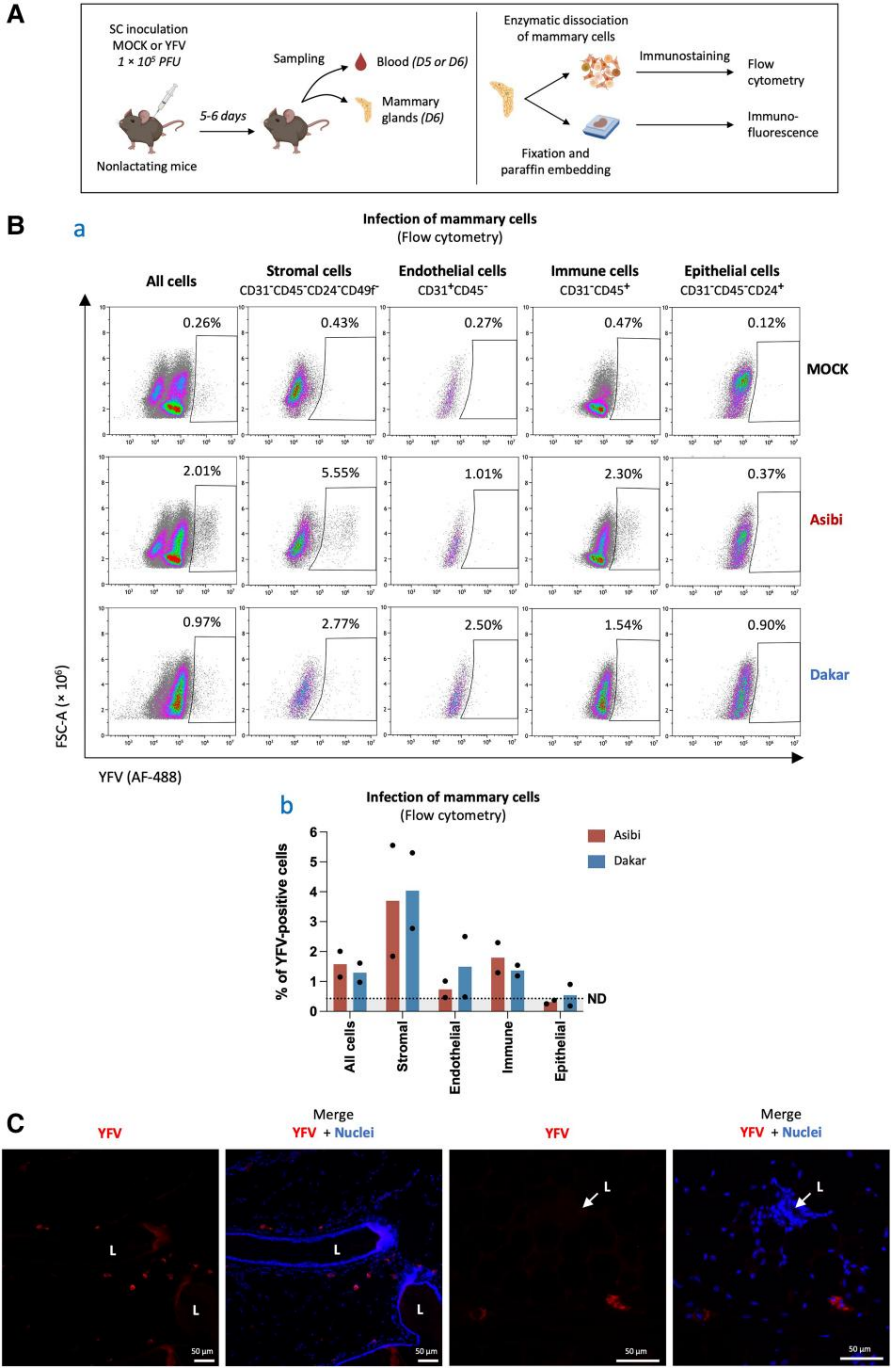
Yellow fever virus (YFV) infects mainly immune and stromal cells in the mammary glands of nonlactating mice in vivo*. A*, Nonlactating A129 mice (11–19 weeks old) were either MOCK-inoculated or inoculated with YFV via subcutaneous (SC) injection of 1 × 10^5^ plaque-forming units (PFU) of the Asibi or Dakar strains of YFV. At 5–6 days after infection (D5–D6), blood and thoracic and/or abdominal mammary samples were collected to assess mice infection and viral tropism in the mammary tissue. Mammary glands were analyzed by flow cytometry following enzymatic dissociation and immunostaining, (2 independent experiments; MOCK n = 7, Asibi n = 7, Dakar n = 5) or by immunohistochemistry after fixation, paraffin embedding, sectioning, and immunostaining (2 independent experiments; MOCK n = 1, Asibi n = 4) (illustration created with BioRender). *B*, For flow cytometry, thoracic and abdominal mammary glands from 3–4 mice per group were pooled and enzymatically digested. Cell suspensions were stained with fluorochrome-conjugated antibodies against CD31 (PE), CD45 (APC), CD24 (BV421), and CD49f (PECy7), then fixed, permeabilized, and stained for YFV using mouse polyclonal antibodies (ascitic fluid) and an AF488-conjugated secondary antibody. Samples were analyzed by flow cytometry. Panel (a) shows representative plots; panel (b) quantifies YFV^+^ cells (2 independent experiments; 1 dot = 1 experiment). The dashed line indicates the limit of specificity (determined from MOCK control conditions), below which values were considered as nonspecific (ND). *C*, For immunohistochemistry, mammary glands from Asibi-infected mice were fixed in 10% formalin, paraffin-embedded, sectioned (5 μm), and stained for YFV using the same primary antibody and an AF546-conjugated secondary. Fluorescence microscopy was used for analysis. Red, YFV; blue, nuclei (DAPI). L: lumen of lactiferous ducts, lined by mammary epithelium and surrounded by stroma. Both images are from the same animal and are representative of 4 independent animals.

The mammary gland comprises epithelial structures within a fibro-adipose matrix, featuring various cell types. Stromal cells (including adipocytes and fibroblasts) provide structural support, and endothelial and immune cells manage vascularization and immune surveillance [20]. The pseudostratified epithelium includes luminal cells (milk secretion) and contractile myoepithelial cells (milk ejection). YFV presence in breast milk suggests mammary epithelium viral crossing, potentially through infected immune cell transmigration or epithelial cell infection followed by viral release into the milk or shedding of infected cells. To study YFV tropism, mice were subcutaneously inoculated with YFV (Asibi and Dakar strains at 1 × 10^5^ PFU). Infection was confirmed in plasma (Supplementary Figure 3*A*), and mammary glands were analyzed at 6 dpi using enzymatic dissociation and flow cytometry or immunohistochemistry (Figure 3*A*). Mammary cells were categorized into endothelial cells (CD31^+^), immune cells (CD45^+^), epithelial cells (CD24^+^), and stromal cells (CD31^−^CD45^−^CD24^−^CD49f^−^). Luminal cells (CD24^high^CD49f^low^) and myoepithelial cells (CD24^low^CD49f^high^) were further identified within the epithelial subset (Supplementary Figure 3*B*). Flow cytometry revealed YFV infection in a small proportion of mammary cells, predominantly in stromal and immune cells (approximately 4% and 1.5% of infected cells, respectively), with minimal detection in endothelial and epithelial cells (Figure 3*B*, Supplementary Figure. 3*C*). Immunohistochemistry of mammary glands confirmed presence of YFV-positive cells mainly in the stromal compartment, likely infiltrating immune cells or resident stromal cells (Figure 3*C*). Furthermore, histological analysis of mammary glands from Asibi-infected mice revealed the presence of cellular infiltrates in periductal stromal regions, compatible with a leukocytic infiltration and local inflammatory response (Supplementary Figure 4). These findings identify mammary stromal and immune cells as possible primary YFV targets in vivo, suggesting that infected immune cell transmigration may facilitate viral crossing of the mammary epithelium. Nevertheless, direct epithelial cell infection cannot be excluded, as it might occur below detection thresholds or be influenced by technical limitations, as being among the most difficult cells to dissociate. Moreover, epithelial cells constitute the mammary barrier and are predominant in human and murine milk (up to 98% and 88% of milk cells, respectively [18, 21]), and, although rare, their infection could represent an alternative route for viral passage through the mammary epithelium. To investigate this possibility, we performed in vitro experiments showing that human primary mammary epithelial cells (Supplementary Figure 5*B*) and 2 human mammary epithelial cell lines—luminal MCF7 and myoepithelial MDA-MB-231 (Supplementary Figure 5*C*)—support productive YFV replication for both wild-type and vaccine strains. These results suggest the possibility that the mammary epithelium may contribute to viral excretion into breast milk, either through free virus or infected cell shedding.

### Oral Inoculation of A129 Mouse Pups With YFV Results in Systemic Infection and Can Lead to Neuroinvasion

**Figure 4.**
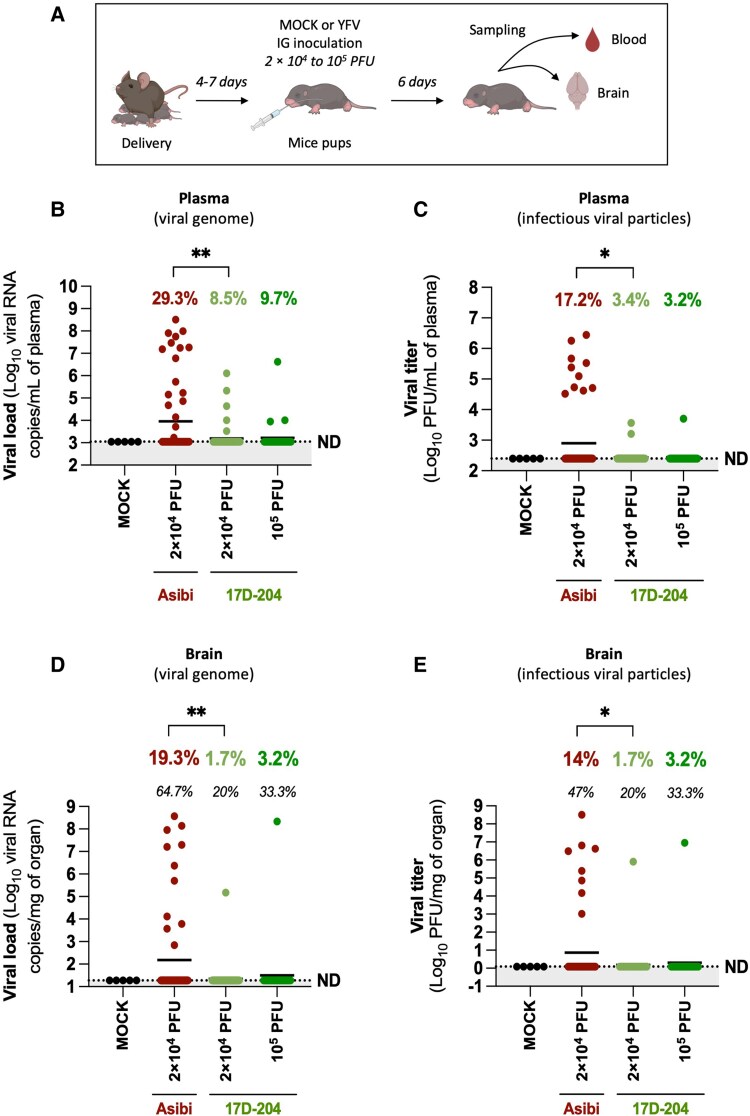
Oral inoculation of A129 mouse pups with yellow fever virus (YFV) results in systemic infection and can lead to neuroinvasion. *A*, A129 mouse pups (4–7 days old) were inoculated via the intragastric (IG) route with YFV or with PBS as a negative control (MOCK). Pups received either phosphate-buffered saline (MOCK, 2 litters, n = 5), 2 × 10^4^ plaque-forming units (PFU) of the Asibi strain (10 litters, n = 58), or 2 × 10^4^ PFU (11 litters, n = 59) or 1 × 10^5^ PFU (4 litters, n = 31) of the 17D-204 strain of YFV (6 independent experiments). Blood and brain samples were collected at 6 days postinfection to assess viremia (*B*, *C*) and brain infection (*D*, *E*) (illustration created with BioRender). *B* and *C*, Viral infection in mouse pups was assessed in plasma (obtained by blood centrifugation) using NS3-specific reverse-transcription quantitative polymerase chain reaction (RT-qPCR) to quantify viral RNA load (*B*), and by plaque assay on Vero cells to determine infectious viral titers (*C*). *D* and *E*, YFV presence in brain tissue was evaluated posthomogenization by RT-qPCR for viral RNA detection (*D*), and by plaque assay on Vero cells to quantify infectious virus (*E*). Results are presented as mean values. The dashed lines indicate the limit of detection or specificity (determined from MOCK control conditions), below which values were considered nonspecific or not detected. The highest of these 2 thresholds was chosen as the final limit (ND). Percentages in bold (*B–E*) represent the proportion of positive samples out of the total; percentages in italic (*D*, *E*) represent the proportion of brain-positive samples among those positive in plasma by RT-qPCR. Statistical test: Fisher exact test (*B–E*). For statistical analyses: *P < .05; **P < .005. Only statistically significant differences (*P* < .05) are indicated.

After excretion in milk, YFV must cross the infant's digestive barrier (tonsils, intestinal epithelium, etc) to allow transmission via breastfeeding. To assess potential oral transmission of YFV, susceptibility of 4- to 7-day-old mouse pups after intragastric inoculation with wild-type (Asibi) or vaccine (17D-204) strains (2 × 10^4^ to 1 × 10^5^ PFU) was evaluated. At 6 dpi, blood and brain samples were collected to evaluate systemic infection and neuroinvasion (Figure 4*A*). Plasma vRNA was detected for both strains, with significantly higher infection rates for Asibi (29.3%) than for 17D-204 at either viral dose (8.5% and 9.7%, respectively) (Figure 4*B*). Infectious virus was found in pups with high plasma viremia (>5 × 10^5^ vRNA copies/mL), titers being higher for Asibi (up to 2.8 × 10^6^ PFU/mL) than for 17D-204 (up to 3.7 × 10^3^ PFU/mL) (Figure 4*C*). Neuroinvasion occurred for both strains. In Asibi-infected pups, vRNA and infectious virus were detected in the brain of 64.7% and 47% of plasma-positive animals, respectively (Figure 4*D* and 4*E*). For 17D-204, brain infection was observed in 1 of 5 and 1 of 3 plasma-positive pups at 2 × 10^4^ and 1 × 10^5^ PFU, respectively, though the limited sample size precludes conclusions on neuroinvasion efficiency. These findings demonstrate that oral YFV inoculation can lead to systemic infection and neuroinvasion in some pups, supporting the plausibility of breastfeeding-associated transmission and neurological complications observed in human cases involving YFV vaccine strains [9–12].

### YFV Can Infect and Cross an In Vitro Model of Human Intestinal Epithelium Without Disrupting Its Integrity

**Figure 5.**
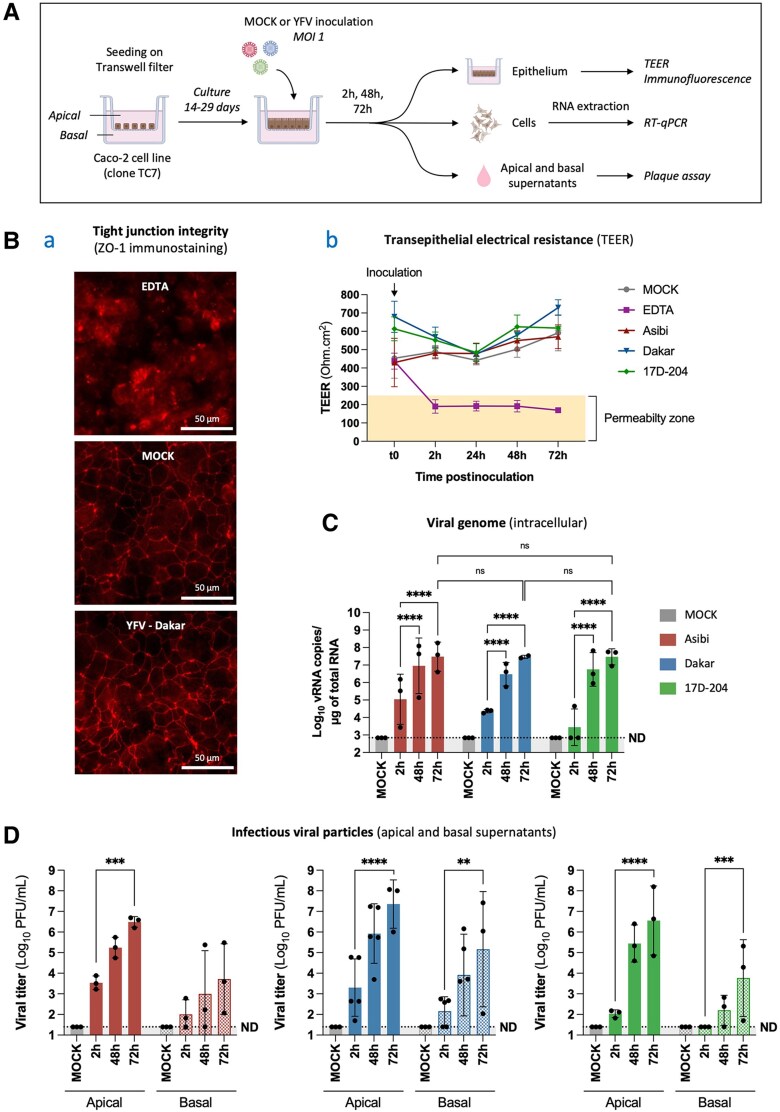
Yellow fever virus (YFV) can infect and cross an in vitro model of human intestinal epithelium without disrupting its integrity. *A*, Caco-2/TC7 cells (3 × 10^4^) were seeded into the upper chamber of Transwell inserts (12 mm diameter, 3 μm pores) and cultured for 14–29 days to form a tight, polarized intestinal epithelial monolayer separating apical (upper) and basolateral (lower) compartments. Monolayer integrity was assessed by transepithelial electrical resistance (TEER) measurements. Cells were apically infected with YFV strains (Asibi, Dakar/HD1279 or 17D-204) at multiplicity of infection (MOI) 1, or left uninfected as controls (MOCK). At various time points postinfection, TEER and ZO-1 immunostaining were used to monitor barrier integrity (B), viral replication was quantified by reverse-transcription quantitative polymerase chain reaction (RT-qPCR) on intracellular RNA (*C*), and viral production and crossing of the epithelium was assessed by plaque assay on apical and basolateral supernatants (*D*) (illustration created with BioRender). *B*, Panel a: At 48 hours postinoculation, monolayer morphology was visualized by immunofluorescence of the tight junction protein ZO-1 (red). Ethylenediaminetetraacetic acid (EDTA; 12.5 mM; purple) was used as a positive control to disrupt barrier function. Panel b: TEER values were monitored over time. The yellow zone (0–250 Ω.cm^2^) indicates the permeability threshold. Data shown are representative of 2 independent experiments and consistent across all replicates. *C*, Viral RNA levels were measured at 2, 48, and 72 hours postinfection. RNA was extracted from Caco-2/TC7 cells, and YFV RNA was quantified using NS3-specific RT-qPCR. Results are expressed as viral RNA copies/μg of total RNA, calculated using a standard curve generated from 10-fold serial dilutions of a quantified YFV NS3 plasmid. *D*, At 2, 48, and 72 hours postinfection, apical and basolateral supernatants were collected and infectious virus (plaque-forming units [PFU]) was quantified by plaque assay on Vero cells. Results in (*A*, *C*, and *D*) are expressed as mean ± standard deviation. The dashed lines indicate the limit of detection or specificity (determined from MOCK control conditions), below which values were considered nonspecific or not detected. The highest of these 2 thresholds was chosen as the final limit (ND). Data in panels (*C*) and (*D*) are representative of minimum 3 independent experiments, with each dot corresponding to the mean value from 1 experiment. Statistical test: Ordinary 2-way ANOVA followed by Tukey multiple comparisons test, performed on log_10_-transformed data (*C*) and ordinary one-way ANOVA followed by Tukey multiple comparisons test, performed separately for strain and apical/basal supernatants and on log_10_-transformed data (using all individual data points, not only experiment means) (*D*). Statistical analyses: **P < .005; ***P < .0005; ****P < .0001; ns, not significant.

Oral infection suggests that YFV can cross a digestive barrier, such as the tonsillar or intestinal mucosa. To investigate whether the intestinal epithelium could allow YFV entry following oral exposure, a previously established in vitro model of human intestinal epithelium based on Caco-2/TC7 cells cultured on Transwell inserts was used. After 14–29 days, tight polarized monolayers (transepithelial electrical resistance [TEER] >250 Ω.cm^2^) were apically inoculated at multiplicity of infection 1 with YFV (Asibi, Dakar, 17D-204 strains). Barrier integrity was monitored by TEER measurement and tight junction protein Zonula Occludens-1 (ZO-1) immunostaining. Intracellular RT-qPCR and plaque assays of apical and basolateral media were used to assess viral replication, production, and epithelial crossing (Figure 5*A*). ZO-1 immunoreactivity at 48 hours postinfection (hpi) revealed intact tight junctions in YFV-infected monolayers (Figure 5*B*-a). Similarly, TEER values remained stable in YFV-infected cells, contrary to ethylenediaminetetraacetic acid–treated controls (Figure 5*B*-b), indicating preserved barrier integrity. Intracellular vRNA increased significantly over time for all strains, peaking at 72 hpi (∼3 × 10^7^ copies/µg) and confirming YFV replication in differentiated enterocytes (Figure 5*C*). Infectious titers showed significant increase apically and basolaterally for all strains, though the increase for Asibi on the basolateral side was not statistically significant (Figure 5*D*). Basolateral titers reached high levels by 72 hpi (Asibi: 5.1 × 10^3^, Dakar: 1.5 × 10^6^, 17D-204: 5.9 × 10^3^ PFU/mL), supporting productive infection in the basolateral compartment, though passive transcytosis of viral particles cannot be excluded. These results support the intestinal epithelium as a potential portal of entry for both wild-type and vaccine YFV strains via productive infection of enterocytes.

### Limited but Detectable Transmission of YFV to Suckling Pups Through Breastfeeding

**Figure 6.**
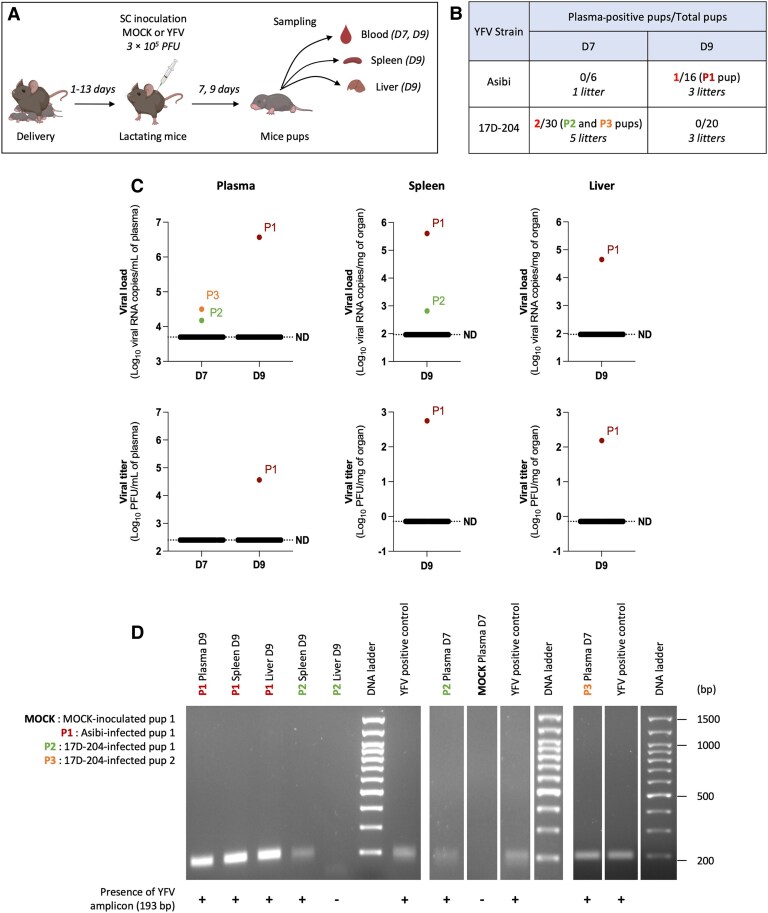
Limited but detectable transmission of yellow fever virus (YFV) to suckling pups through breastfeeding. *A*, Lactating A129 mice (12–17 weeks old) were inoculated subcutaneously (SC) 1–13 days postpartum with either phosphate-buffered saline (MOCK, n = 4) or 3 × 10^5^ plaque-forming units (PFU) of Asibi (n = 3) or 17D-204 (n = 5) strains of YFV (3 independent experiments). Blood, spleen, and liver samples were collected from suckling pups at 7 (D7) and/or 9 (D9) days postinfection to assess transmission and viral dissemination (Asibi n = 16, 3 litters; 17D-204 n = 20, 5 litters) (illustration created with BioRender). *B*, Viral RNA levels in pup plasma were quantified by NS3-specific reverse-transcription quantitative polymerase chain reaction (RT-qPCR). The same pups were analyzed at both time points: The 6 Asibi-inoculated pups sampled at 7 dpi are part of the 16 analyzed at 9 dpi, and the 20 pups inoculated with 17D-204 at 9 dpi are included in the 30 analyzed at 7 dpi. *C*, Viral RNA and infectious titers in plasma, spleen, and liver were measured at 7 (D7) and 9 (D9) days postinfection in breastfed pups from dams infected with Asibi or 17D-204 (data shown on the same x-axis). Organs were homogenized prior to analysis by RT-qPCR and plaque assay. P1 (4–5 days old), P2 (7–10 days old), and P3 (4 days old) represent the 3 plasma-positive pups shown in (*B*). The dashed lines indicate the limit of detection or specificity (determined from MOCK control conditions), below which values were considered nonspecific or not detected. The highest of these 2 thresholds was chosen as the final limit (ND). *D*, In pups with plasma positive for YFV (C), the presence of viral RNA in spleen and liver was further evaluated by RT-qPCR following tissue homogenization. RT-qPCR–positive samples were then subjected to electrophoresis on a 2% Tris-Acetate-EDTA agarose gel to confirm amplification of the expected viral amplicons. A 100 bp Plus DNA ladder was used to estimate fragment size, with the expected amplicon corresponding to 193 bp. Negative control included qPCR products from mock-infected pup samples (MOCK), while a plasmid containing YFV sequence (equivalent to 4 viral RNA copies/µL) served as a positive control (YFV-positive control). Full-size agarose gel electrophoresis images are shown in Supplementary Figure 4. MOCK, MOCK-inoculated pup 1; P1, Asibi-infected pup 1; P2, 17D-204-infected pup 1; P3, 17D-204-infected pup 2.

After validating the sequential steps of milk-borne YFV transmission, breastfeeding transmission experiments were performed in our mouse model to confirm this route of infection. Lactating A129 mice nursing 1- to 13-day-old pups were inoculated postpartum with YFV (Asibi and 17D-204 strains) at 3 × 10^5^ PFU. At 7 and/or 9 dpi, samples from suckling pups (blood, spleen, liver) were collected to assess infection (Figure 6*A*). Dams’ infection was confirmed by viremia (Supplementary Figure 6*A*). Among all pups, 3 tested positive for YFV RNA in plasma by RT-qPCR—1 of 16 fed by an Asibi-infected dam (P1, 4–5 days old) and 2 of 30 by 17D-204–infected dams (P2, 7–10 days old; P3, 4 days old) (Figure 6*B*)—demonstrating that YFV breastfeeding transmission can occur, albeit infrequently. Infected pups were further analyzed on plasma, spleen, and liver by RT-qPCR and plaque assay (Figure 6*C*), with confirmation of RT-qPCR amplicons by gel electrophoresis (Figure 6*D*). P1 showed high viral loads and infectious titers in all tissues at 9 dpi, with detection of the YFV-specific 193 bp amplicon, confirming systemic infection via breastfeeding. P2 and P3 had lower plasma RNA levels at 7 dpi; and at 9 dpi, only P2 showed low vRNA in the spleen and a positive viral amplicon. These results demonstrate that both wild-type and vaccine YFV strains can be transmitted through breastfeeding in mice, although such transmission appears to be a rare event.

## DISCUSSION

Despite the availability of an effective live-attenuated vaccine, YFV remains a major public health concern. While mosquito transmission is primary, cases of vaccine-strain YFV transmission via breastfeeding, leading to meningoencephalitis in infants [9–12], have prompted updated recommendations [13]. Detection of wild-type YFV RNA in human breast milk during outbreaks further raised concerns about potential nonvectorial transmission [14]. However, confirming this risk in endemic settings is difficult due to constant mosquito exposure. To address this, we conducted the first proof-of-concept study using A129 mice to investigate YFV transmission through breastfeeding and characterize underlying mechanisms.

We showed that both wild-type and vaccine YFV strains reach mammary glands in mice and are shed in breast milk at similar titers, though the vaccine strain disseminated to fewer glands. At 5–6 dpi, infectious virus was detected in 38.5% (Asibi) and 23.1% (17D-204) of lactating mice—less frequently than in nonlactating mice (82.8% and 42.9%, respectively), possibly due to milk presence and physiological changes affecting detection. Infectious virus of both YFV strains was detected in breast milk, whey, and cell fraction, indicating the presence of free viral particles and cell-associated infectious virus. Despite reduced mammary dissemination, the vaccine strain reaches breast milk at levels similar to the wild-type strain, suggesting different mammary crossing mechanisms. In vivo, YFV primarily targeted mammary stromal and immune cells; however, mammary epithelial cells are also permissive in vitro. These findings suggest 2 potential routes into milk: direct epithelial infection with viral release or shedding, and infected immune cells transmigration (“Trojan horse” strategy), although other mechanisms cannot be excluded. Elucidating these mechanisms remains challenging and poorly defined, even for established breastfeeding-transmitted viruses like HIV, HTLV-1, and CMV [17]. Organoid models may help clarify epithelial susceptibility but may not fully recapitulate dynamics required to study viral crossing mechanisms [22].

Crucially, both strains infected pups after oral exposure, with higher rates for Asibi (29.3%) than 17D-204 (8.5%–9.7%), highlighting oral YFV transmission. Neuroinvasion occurred with both strains, especially Asibi (64.7% of infected pups). Together with reported neurological symptoms in cases of breastfeeding transmission of vaccine strains in humans [9–12], our findings raise concerns about the neurovirulence of wild-type YFV following oral exposure. Using a human intestinal epithelium model, we found that differentiated enterocytes support productive viral replication, and apical-to-basal viral crossing in vitro. While transcytosis cannot be ruled out, paracellular passage seems unlikely given preserved barrier integrity. Finally, as proof of concept, we confirmed rare but detectable YFV transmission through breastfeeding to suckling pups in vivo.

Overall, our findings highlight phenotypic differences between wild-type and vaccine YFV strains in dissemination and transmission efficiency. Both reach the mammary glands and are shed in milk at similar titers, despite less frequent gland infection with the vaccine strain. Oral infection experiments suggest higher neonatal susceptibility to the wild-type strain, indicating strain-specific differences in epithelial barriers crossing. These differences may be linked to the 20 amino acid differences, mainly in the envelope protein, between Asibi and 17D [4], which may influence entry pathways and barrier-crossing mechanism.

Mice are not natural hosts and are naturally resistant to YFV infection except when extremely young or infected intracranially [23–26]. Therefore, the A129 model, lacking the type I interferon receptor, is widely used, despite its inherent limitations for studying YFV pathogenesis and immune responses, as absence of type I interferon signaling can enhance viral replication and dissemination. Some aspects of infection such as neuroinvasion or tissue tropism may be more pronounced than in immunocompetent hosts, and direct extrapolation to human infection should be made with caution. Nevertheless, the A129 model remains biologically relevant [19], as YFV itself partially inhibits interferon signaling in humans—but not in mice—through NS5-mediated STAT2 degradation. Importantly, it provides a sensitive and discriminative system to compare viral strains in vivo and to assess antiviral or vaccine efficacy under controlled conditions [27–32]. This discriminative capacity is further supported by the present findings on YFV and by our previous work on ZIKV transmission in the same model. ZIKV, another orthoflavivirus, was transmitted via breastfeeding in A129 mice with markedly higher efficiency (39%–90%) [18, 33] than YFV (∼6%), reaching substantially higher milk titers (up to 10⁹ vs 1.5 × 10⁵ PFU/mL) [18] and showing greater oral infectivity (64% vs 29.3% and 8.5% for wild-type and vaccine YFV, respectively) [33]. These findings suggest a markedly greater lactogenic transmission potential for ZIKV and emphasize the biological relevance of the A129 model for studying milk-borne transmission efficacy of orthoflaviviruses.

Breastfeeding remains essential for infant health and is still recommended in ZIKV-endemic areas [34]. However, with confirmed vaccine-strain YFV transmission via breastfeeding in humans [9–12], our findings reinforce the need to include this route among potential transmission risks for both YFV and ZIKV and to reassess current public health recommendations, especially in the absence of clear guidelines during maternal infection.

## Supplementary Material

jiaf637_Supplementary_Data

## Supplementary Data

Supplementary materials are available at *The Journal of Infectious Diseases* online (http://jid.oxfordjournals.org/). Supplementary materials consist of data provided by the author that are published to benefit the reader. The posted materials are not copyedited. The contents of all supplementary data are the sole responsibility of the authors. Questions or messages regarding errors should be addressed to the author.

## References

[1] World Health Organization Regional Office for the Eastern Mediterranean. Yellow fever. Available at: http://www.emro.who.int/health-topics/yellow-fever/index.html. Accessed 12 May 2025.

[2] Monath TP, Vasconcelos PF. Yellow fever. J Clin Virol. 2015; 64:160–73. 25453327 10.1016/j.jcv.2014.08.030

[3] Tuells J, Henao-Martínez AF, Franco-Paredes C. Yellow fever: a perennial threat. Arch Med Res 2022; 53:649–57. 36404585 10.1016/j.arcmed.2022.10.005

[4] Monath TP . Yellow fever vaccine. Expert Rev Vaccines 2005; 4:553–74. 16117712 10.1586/14760584.4.4.553

[5] Centers for Disease Control and Prevention . Transfusion-related transmission of yellow fever vaccine virus—California, 2009. MMWR Morb Mortal Wkly Rep 2010; 59:34–7. 20094025

[6] Gould CV, Free RJ, Bhatnagar J, et al Transmission of yellow fever vaccine virus through blood transfusion and organ transplantation in the USA in 2021: report of an investigation. Lancet Microbe 2023; 4:e711–21. 37544313 10.1016/S2666-5247(23)00170-2 PMC11089990

[7] Bentlin MR, de Barros Almeida RAM, Coelho KIR, et al Perinatal transmission of yellow fever, Brazil, 2009. Emerg Infect Dis 2011; 17:1779. 21888828 10.3201/eid1709.110242 PMC3322086

[8] Tsai T, Paul R, Lynberg M, Letson G. Congenital yellow fever virus infection after immunization in pregnancy. J Infect Dis 1993; 168:1520–3. 8245539 10.1093/infdis/168.6.1520

[9] Centers for Disease Control and Prevention . Transmission of yellow fever vaccine virus through breast-feeding—Brazil, 2009. MMWR Morb Mortal Wkly Rep 2010; 59:130–2. 20150888

[10] Kuhn S, Twele-Montecinos L, MacDonald J, Webster P, Law B. Case report: probable transmission of vaccine strain of yellow fever virus to an infant via breast milk. CMAJ 2011; 183:E243–5. 21324845 10.1503/cmaj.100619 PMC3050973

[11] Traiber C, Coelho-Amaral P, Ritter VRF, Winge A. Infant meningoencephalitis caused by yellow fever vaccine virus transmitted via breastmilk. J Pediatr (Rio J) 2011; 87:269–72. 21461453 10.2223/JPED.2067

[12] World Health Organization . Global Advisory Committee on Vaccine Safety, 16–17 June 2010. Wkly Epidemiol Rec 2010; 85:285–91.

[13] Centers for Disease Control and Prevention . Yellow fever vaccine: recommendations of the Advisory Committee on Immunization Practices (ACIP). MMWR Recomm Rep 2010; 59(RR-7):1–27. 20671663

[14] Ribeiro AF, de Resende Brasil LMC, Prada RM, Nogueira JS, Maeda AY, Sztajnbok J. Detection of wild-type yellow fever virus in breast milk. Pediatr Infect Dis J 2020; 39:68–9. 31725551 10.1097/INF.0000000000002496

[15] Van de Perre P, Molès J, Nagot N, et al Revisiting Koch's postulate to determine the plausibility of viral transmission by human milk. Pediatr Allergy Immunol 2021; 32:835–42. 33594740 10.1111/pai.13473 PMC8359252

[16] Whitman L. Failure of *Aedes aegypti* to transmit yellow fever cultured virus (17D). Am J Trop Med Hyg 1939; s1-19 : Issue 1:19–26.

[17] Desgraupes S, Hubert M, Gessain A, Ceccaldi P-E, Vidy A. Mother-to-child transmission of arboviruses during breastfeeding: from epidemiology to cellular mechanisms. Viruses 2021; 13:1312. 34372518 10.3390/v13071312 PMC8310101

[18] Desgraupes S, Jeannin P, Gessain A, Ceccaldi P-E, Vidy A. Excretion of cell-free and cell-associated Zika virus into breast milk of infected dams and identification of antiviral factors. Viruses 2022; 14:851. 35632593 10.3390/v14050851 PMC9144681

[19] Meier KC, Gardner CL, Khoretonenko MV, Klimstra WB, Ryman KD. A mouse model for studying viscerotropic disease caused by yellow fever virus infection. PLoS Pathog 2009; 5:e1000614. 19816561 10.1371/journal.ppat.1000614 PMC2749449

[20] Inman JL, Robertson C, Mott JD, Bissell MJ. Mammary gland development: cell fate specification, stem cells and the microenvironment. Development 2015; 142:1028–42. 25758218 10.1242/dev.087643

[21] Witkowska-Zimny M, Kaminska-El-Hassan E. Cells of human breast milk. Cell Mol Biol Lett 2017; 22:1–11. 28717367 10.1186/s11658-017-0042-4 PMC5508878

[22] Sumbal J, Chiche A, Charifou E, Koledova Z, Li H. Primary mammary organoid model of lactation and involution. Front Cell Dev Biol 2020; 8:68. 32266252 10.3389/fcell.2020.00068 PMC7098375

[23] Fitzgeorge R, Bradish C. The in vivo differentiation of strains of yellow fever virus in mice. J Gen Virol. 1980; 46:1–13. 6766176 10.1099/0022-1317-46-1-1

[24] Theiler M . Susceptibility of white mice to the virus of yellow fever. Science 1930; 71:367. 10.1126/science.71.1840.367 17731835

[25] Liprandi F . Isolation of plaque variants differing in virulence from the 17D strain of yellow fever virus. J Gen Virol 1981; 56:363–70. 7310380 10.1099/0022-1317-56-2-363

[26] Barrett AD, Gould EA. Comparison of neurovirulence of different strains of yellow fever virus in mice. J Gen Virol 1986; 67:631–7. 3958694 10.1099/0022-1317-67-4-631

[27] Jiang X, Dalebout TJ, Lukashevich IS, Bredenbeek PJ, Franco D. Molecular and immunological characterization of a DNA-launched yellow fever virus 17D infectious clone. J Gen Virol 2015; 96:804–14. 25516543 10.1099/jgv.0.000026 PMC4811652

[28] de Freitas CS, Higa LM, Sacramento CQ, et al Yellow fever virus is susceptible to sofosbuvir both in vitro and in vivo. PLoS Negl Trop Dis 2019; 13:e0007072. 30699122 10.1371/journal.pntd.0007072 PMC6375661

[29] Piras-Douce F, Raynal F, Raquin A, et al Next generation live-attenuated yellow fever vaccine candidate: safety and immuno-efficacy in small animal models. Vaccine 2021; 39:1846–56. 33685778 10.1016/j.vaccine.2021.02.033 PMC8047865

[30] Qian X, Wu B, Tang H, et al Rifapentine is an entry and replication inhibitor against yellow fever virus both in vitro and in vivo. Emerg Microbes Infect 2022; 11:873–84. 35249454 10.1080/22221751.2022.2049983 PMC8942558

[31] Guo W, Jiang T, Rao J, et al A safer cell-based yellow fever live attenuated vaccine protects mice against YFV infection. iScience 2024; 27.:110972. 39398246 10.1016/j.isci.2024.110972 PMC11470684

[32] LeCher JC, Costa VV, Rust LN, et al Combating yellow fever virus with 7-deaza-7-fluoro-2′-C-methyladenosine. Antimicrob Agents Chemother 2025; 69:e01889–24. 40227063 10.1128/aac.01889-24 PMC12057363

[33] Hubert M, Jeannin P, Burlaud-Gaillard J, et al Evidence that Zika virus is transmitted by breastfeeding to newborn A129 (Ifnar1 knock-out) mice and is able to infect and cross a tight monolayer of human intestinal epithelial cells. Front Microbiol 2020; 11:524678. 33193119 10.3389/fmicb.2020.524678 PMC7649816

[34] World Health Organization . Guideline: infant feeding in areas of Zika virus transmission. Geneva, Switzerland: World Health Organization, 2021.

